# Extensive Myelinated Retinal Nerve Fibers Associated With Severe Ipsilateral Myopia and Amblyopia: A Case Report

**DOI:** 10.7759/cureus.117740

**Published:** 2026-10-10

**Authors:** Youssef Chajire, Zakaria Azemour, Nabil Bouslous, Omar Moustaine

**Affiliations:** 1 Ophthalmology, Centre Hospitalier Universitaire (CHU) Mohammed VI, Agadir, MAR

**Keywords:** amblyopia, anisometropia, myelinated retinal nerve fiber layers, myopia, ophthalmology

## Abstract

Myelinated retinal nerve fibers are an uncommon congenital retinal anomaly that is usually benign and discovered incidentally. When extensive, however, they may be associated with severe ipsilateral myopia, anisometropia, and amblyopia with limited visual potential. We report the case of a 20-year-old woman with longstanding decreased visual acuity in the right eye. Ophthalmological examination revealed extensive peripapillary myelinated retinal nerve fibers extending across the posterior pole, associated with severe ipsilateral myopia of -18.50 diopters and profound amblyopia. Best-corrected visual acuity was counting fingers at 3 m in the right eye and 20/20 in the left eye. Optical coherence tomography confirmed hyperreflective thickening corresponding to the myelinated retinal nerve fibers and showed marked myopic changes. The combination of extensive myelination, severe anisometropia, and longstanding amblyopia represents an unfavorable prognostic profile for visual recovery. Early recognition, accurate refractive correction, and timely amblyopia management during childhood are essential to maximize visual potential.

## Introduction

Myelinated retinal nerve fibers are a developmental anomaly resulting from the persistence of myelination anterior to the lamina cribrosa [1]. Under normal development, retinal ganglion cell axons become myelinated posterior to the lamina cribrosa, whereas the retinal nerve fiber layer remains unmyelinated [2]. Myelinated retinal nerve fibers typically appear as white or gray-white, striated lesions with feathery margins that follow the distribution of the retinal nerve fiber layer and are often contiguous with the optic disc [3]. Although relatively uncommon, they have been reported in approximately 1% of eyes in some series [4].

Most cases are asymptomatic and are discovered incidentally [5]. However, extensive peripapillary myelinated retinal nerve fibers may be associated with ipsilateral high myopia, anisometropia, strabismus, and amblyopia [6], a constellation frequently referred to as the syndrome of myelinated retinal nerve fibers, myopia, and amblyopia [7,8]. Visual outcomes in these patients vary widely, ranging from profound refractory amblyopia [9,10] to meaningful visual recovery following early optical correction and occlusion therapy. This variability, alongside emerging evidence linking the condition to structural developmental abnormalities such as foveal hypoplasia, highlights the challenges in predicting visual prognosis.

Despite previous documentation of this syndrome, the precise interplay between extensive myelination, severe axial myopia, and the limits of visual rehabilitation remains incompletely understood, leaving a gap regarding optimal management strategies for atypical presentations. Herein, we report a clinical case of extensive unilateral peripapillary myelinated retinal nerve fibers associated with severe ipsilateral high myopia and profound amblyopia in a young adult, aiming to highlight its clinical, structural, and therapeutic features to contribute to the current literature on this rare entity.

## Case presentation

A 20-year-old woman presented to the Department of Ophthalmology with longstanding decreased visual acuity in the right eye (OD). According to the patient, the visual impairment had been present since childhood. She had worn spectacles intermittently since approximately 10 years of age. There was no reported history of ocular trauma, ocular surgery, or other significant ophthalmological disease.

Ophthalmological examination

In the OD, manifest refraction was -18.50 diopters sphere/DS (-2.25 diopters cylinder/DC × 40°). Best-corrected visual acuity was counting fingers at 3 m. Intraocular pressure was 16 mmHg. The anterior segment was unremarkable, with a clear cornea, deep and quiet anterior chamber, normal iris, and clear crystalline lens. In the OS, refraction was +0.25 DS (-1.50 DC × 165°), with best-corrected visual acuity of 20/20. Intraocular pressure was 14 mmHg. The anterior segment examination was normal. Ocular motility examination showed normal ocular alignment (orthophoria), with no evidence of phoria or tropia. The axial length was 29.71 mm in the OD and 23.39 mm in the OS.

Fundus examination of the OD revealed extensive peripapillary myelinated retinal nerve fibers, appearing as dense, white, opaque, striated lesions with characteristic feathery margins. The myelination was predominantly located around the optic disc and extended toward the posterior pole, partially obscuring the underlying retinal vessels. The optic disc was partially obscured by the extensive myelinated fibers. The fundus also showed features consistent with high myopia. In the OS, the fundus examination was unremarkable (Figure 1).

**Figure 1:**
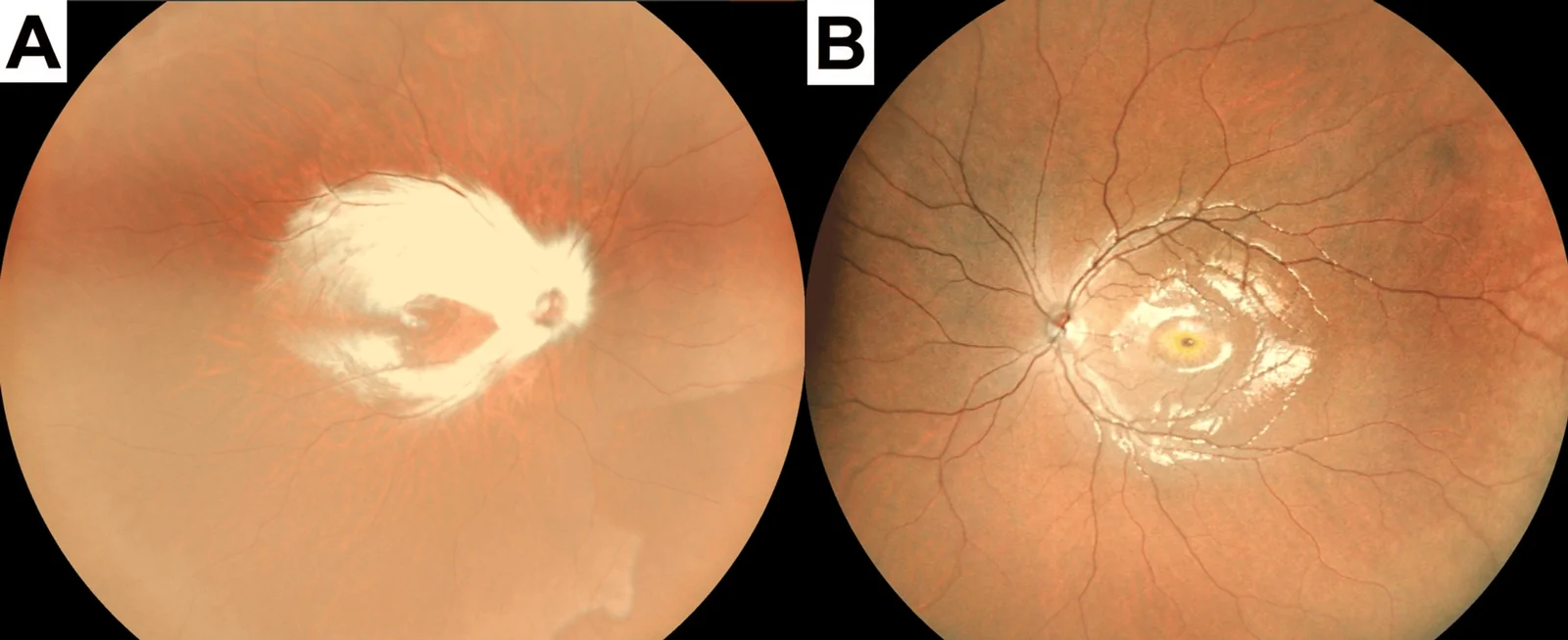
Fundus photograph showing myelinated nerve fibers in the right eye (A) and the normal left eye (B) Fundus photograph showing extensive peripapillary myelinated retinal nerve fibers, appearing as dense, white, opaque, striated lesions with characteristic feathery margins in the right eye (A) and a normal left eye (B).

Spectral-domain optical coherence tomography (SD-OCT) of the OD demonstrated marked hyperreflective thickening of the retinal nerve fiber layer corresponding to the areas of myelination. The macular OCT showed significant myopic structural changes, with posterior pole distortion and marked chorioretinal thinning. No obvious intraretinal or subretinal fluid was identified on the available scan (Figure 2). In the OS, the SD-OCT was normal (Figure 3). Foveolar and paramacular thicknesses were 240 and 420 µm, respectively, in the OD as well as 200 and 350 µm, respectively, in the OS.

**Figure 2:**
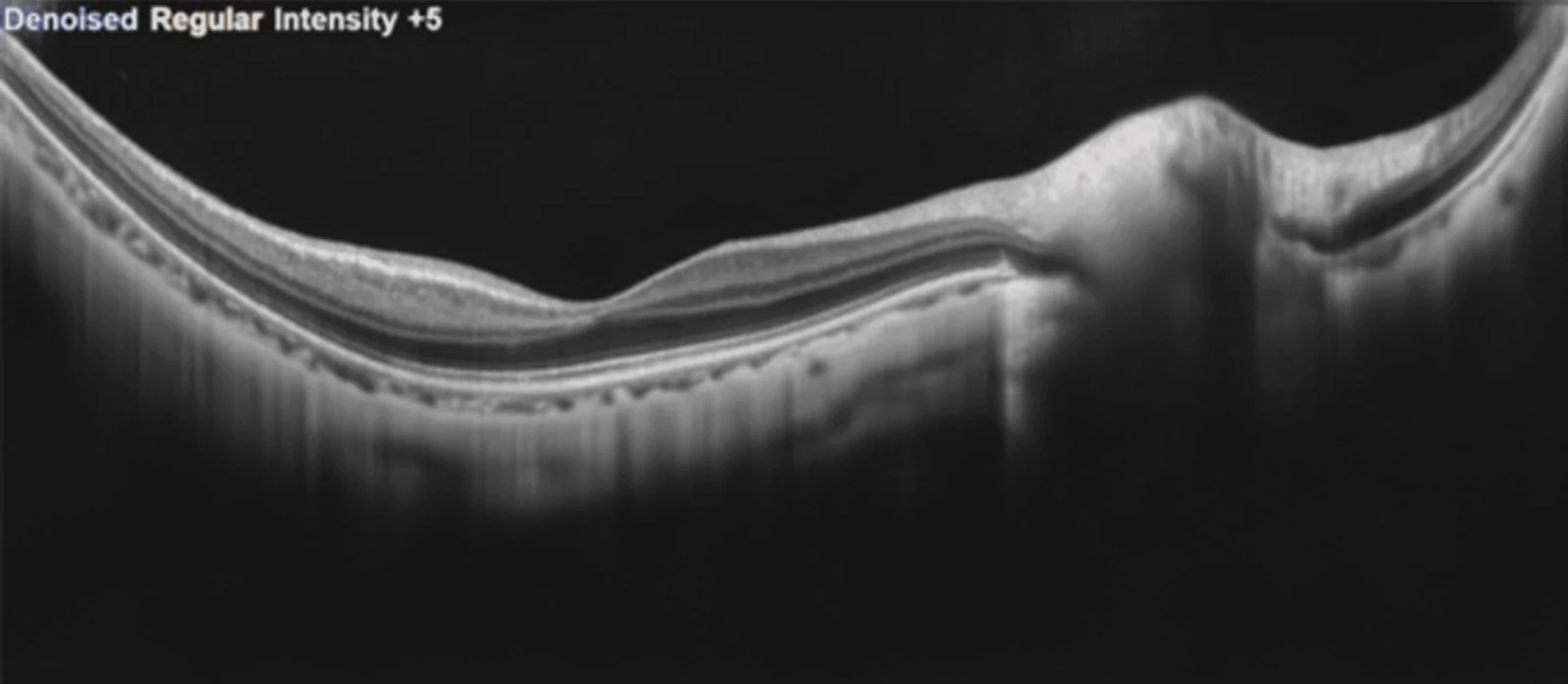
Spectral-domain optical coherence tomography (SD-OCT) of the right eye showing marked hyperreflective thickening of the retinal nerve fiber layer corresponding to the areas of myelination

**Figure 3:**
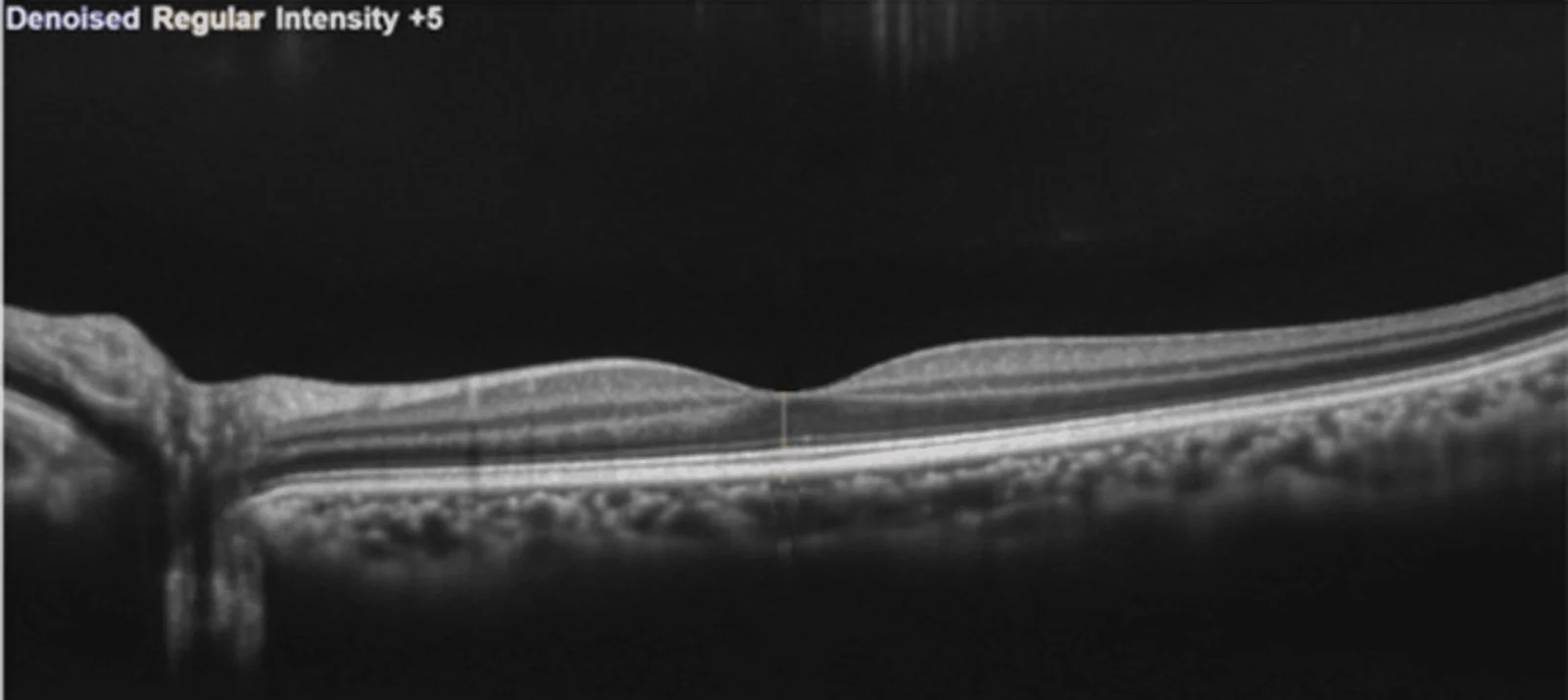
Spectral-domain optical coherence tomography (SD-OCT) of the left eye showing normal retinal nerve fiber layer thickness and a normal macular profile

The marked interocular refractive difference, longstanding visual impairment, and extensive unilateral myelinated retinal nerve fibers were consistent with myelinated retinal nerve fibers, high myopia, and amblyopia syndrome. At presentation, a rigid gas-permeable contact lens trial was performed to assess the potential contribution of corneal optical aberrations to the reduced visual acuity. No improvement in visual acuity was observed with the rigid contact lens.

Treatment and follow-up

The patient was prescribed full optical correction based on cycloplegic refraction and occlusion therapy of the fellow eye for several hours per day. Follow-up examinations were performed at three and six months. Despite good adherence to the prescribed treatment, no improvement in best-corrected visual acuity was observed in the affected eye. The visual acuity remained counting fingers at 3 m at both follow-up visits. The patient was counseled regarding the guarded visual prognosis, given the longstanding profound amblyopia, severe anisometropia, extensive peripapillary myelination, and associated high myopia.

## Discussion

Myelinated retinal nerve fibers are relatively uncommon developmental anomalies, reported in approximately 1% of eyes in some series [1,5]. They result from abnormal persistence of myelinating tissue anterior to the lamina cribrosa. Clinically, the lesions appear as white or gray-white striated patches with feathery borders that follow the distribution of the retinal nerve fiber layer [1,5]. In a large autopsy series of 3,968 cases, Straatsma et al. identified myelinated retinal nerve fibers in 0.98% of cases and 0.54% of eyes, with bilateral involvement in 7.7% of affected cases [1].

The association between extensive myelinated retinal nerve fibers, ipsilateral high myopia, amblyopia, and strabismus has been recognized for several decades. Straatsma et al. first described a characteristic syndrome consisting of extensive unilateral myelinated retinal nerve fibers associated with ipsilateral myopia, amblyopia, and strabismus [6]. Subsequent reports confirmed this association and emphasized the potential for severe visual impairment in affected eyes [5,7]. Hittner and Antoszyk described 12 patients with unilateral peripapillary myelinated nerve fibers associated with myopia and/or amblyopia. Seven patients had greater anisometropia and abnormal macular findings and achieved final visual acuities of 20/200 or worse despite conventional amblyopia therapy, whereas five patients with less anisometropia and normal macular appearance achieved visual acuities of 20/30 or better [2]. These findings suggest that the visual prognosis is influenced not only by the degree of refractive error but also by associated macular abnormalities.

Kee and Hwang subsequently evaluated the visual prognosis of amblyopia associated with anisomyopia and myelinated retinal nerve fibers in 12 children treated with spectacle correction and occlusion therapy [3]. Five patients achieved visual acuities of 20/60 or better, including four who reached 20/30, whereas six remained at 20/200 or worse. Greater anisometropia and a larger area of myelination were associated with poorer visual outcomes [3]. These findings indicate that the presence of myelinated retinal nerve fibers does not invariably imply irreversible visual loss, but the extent of myelination and severity of anisometropia may be important prognostic factors.

Other reports have similarly demonstrated variable responses to amblyopia treatment. Lee and Gonzalez described four patients with unilateral peripapillary myelinated retinal nerve fibers, myopia, strabismus, and amblyopia, all of whom had poor visual outcomes despite full cycloplegic correction and appropriate patching [7]. A Moroccan report by Laghmari et al. described extensive peripapillary myelinated nerve fibers associated with high ipsilateral myopia and refractory amblyopia despite optical correction and intensive occlusion therapy [4]. In contrast, Summers et al. reported two young children with the same clinical association who achieved visual acuities of 20/30 and 20/50 after occlusion therapy, despite abnormal macular reflexes [8]. These contrasting outcomes indicate that amblyopia treatment should not be considered futile solely because extensive myelinated retinal nerve fibers are present, particularly when treatment is initiated early [3,8].

The mechanisms underlying the association between extensive myelinated retinal nerve fibers, high myopia, and amblyopia remain incompletely understood. Proposed explanations include abnormal development of the optic nerve and lamina cribrosa, extensive ectopic myelination, anisometropic refractive error, and associated macular abnormalities [5-7]. The presence of extensive myelination may also interfere with the visualization and assessment of the underlying retinal structures. More recent observations have further suggested that extensive myelinated retinal nerve fibers may be associated with developmental macular abnormalities. Hallali et al. described six patients with extensive myelinated retinal nerve fibers, all of whom had myopia and amblyopia, and demonstrated bilateral foveal hypoplasia on OCT, supporting the concept that this condition may represent part of a broader developmental retinal phenotype [10].

Our patient exhibited several unfavorable prognostic factors, including extensive peripapillary myelination, severe unilateral myopia of −18.50 diopters, marked anisometropia, and profound longstanding visual impairment. The severity of the refractive asymmetry and the extensive distribution of myelination are consistent with factors previously associated with poor visual outcomes [2,3]. The association with high myopia and severe amblyopia is also consistent with the clinical spectrum described in previous reports and reviews [7,9]. The marked visual deficit in our patient may therefore reflect the combined effects of longstanding anisometropic amblyopia and structural abnormalities associated with extensive myelinated retinal nerve fibers and high myopia.

An important clinical implication is the need for early detection and treatment. Children with extensive myelinated retinal nerve fibers should undergo careful cycloplegic refraction, assessment of visual acuity, evaluation for anisometropia and strabismus, and detailed examination of the macula. Early and consistent refractive correction and occlusion therapy may maximize visual development before the end of the critical period [3,4,8]. The variability in treatment response reported in the literature supports attempting appropriate amblyopia therapy rather than assuming that visual loss is irreversible [3,8].

In the present case, the very late presentation at 20 years of age considerably limited the potential for functional recovery. Despite maximum tolerated optical correction following cycloplegic refraction and occlusion of the healthy eye for several hours per day, no improvement in visual acuity was observed at the three- and six-month follow-ups. A rigid contact lens trial performed during the consultation also failed to improve visual acuity. This unfavorable response is consistent with previous reports describing refractory amblyopia in patients with extensive myelinated retinal nerve fibers and severe anisometropic myopia [4,7]. Nevertheless, the favorable responses reported in younger patients emphasize the importance of early recognition and treatment [3,8]. This case illustrates that extensive retinal myelination associated with severe unilateral myopia should prompt careful assessment for amblyopia, anisometropia, and associated macular developmental abnormalities.

## Conclusions

The association of extensive myelinated retinal nerve fibers, severe ipsilateral high myopia, and profound amblyopia represents a rare and complex clinical entity. As illustrated by our case and supported by previous literature, factors such as the extent of myelination, severe anisometropia, and delayed treatment initiation are frequently associated with a guarded visual prognosis. While early recognition and timely management during childhood remain crucial when possible, this case highlights the clinical challenges encountered in late-presenting individuals, where visual recovery may be severely limited, and management primarily focuses on appropriate counseling and monitoring.
